# Nanoparticles reveal Extreme Size-Sorting and Morphologies in Complex Coacervate Superstructures

**DOI:** 10.1038/s41598-018-32240-5

**Published:** 2018-09-14

**Authors:** Jan Bart ten Hove, Matthias N. van Oosterom, Fijs W. B. van Leeuwen, Aldrik H. Velders

**Affiliations:** 1 Laboratory of BioNanoTechnology, Wageningen University, Bornse Weilanden 9, Wageningen, The Netherlands; 2 Interventional Molecular Imaging Laboratory, Department of Radiology, Leiden University Medical Centre, Leiden, The Netherlands

## Abstract

We here provide detailed insight in self-assembled complex coacervate systems exploiting gold nanoparticles for cryoTEM contrast. Nanoparticle-containing dendrimicelles are formed from fifth-generation dendrimer-encapsulated nanoparticles (DENs) and dendrimer-stabilized nanoparticles (DSNs). The complex coacervate structures self-organize in biconcave thin water layers into size-sorted monolayer superstructures. The embedded nanoparticles are a straightforward tool to visualize dendrimicelles and determine the aggregation number and polydispersity. The superstructure shows extreme size-sorting patterns which, contrary to related systems with higher generation dendrimers, consists not only of dendrimicelles but also much bigger complex coacervate nanoassemblies, such as vesicles.

## Introduction

Hierarchical assembly allows for sophisticated and well-defined ordering of molecular building blocks into complex materials that exhibit unique properties^1–3^. Among the many different self-assembled nanomaterials that have been created, e.g, metal-organic frameworks^4^, molecular boxes^5,6^, and vesicles^7^, micelles have proven to be most versatile supramolecular structures^8^. Complex coacervate core micelles, (C3Ms), form upon the combination of oppositely-charged polymeric building blocks and have been formed using a plethora of macromolecules, ranging from proteins to linear-, coordination-, branched-, and hyper-branched polymers^1,9–12^. Well-defined polymers such as dendrimers allow for detailed investigations on structure, composition, shape and stability of C3Ms as we showed before^13^. Dendrimers are known for their ability to encapsulate small molecules and nanoparticles^14,15^. Poly(AmidoAmine), PAMAM, dendrimers are among the most studied dendrimers^16–18^. From the original work of Amis and Crooks, it is known that only the higher generations amine-terminated PAMAM (*i.e*., generations six through nine) can effectively encapsulate a nanoparticle (*i.e*., Au, Pd, Cu, etc.) inside their interior cavities, yielding DENs^14,19^. In fact, PAMAM generations 0–3 tend to form dendrimer-stabilized nanoparticles, DSNs, instead, where a nanoparticle is stabilized by multiple dendrimers rather than being encapsulated in a single, individual, dendrimer. As a result, DSNs are considerably larger than DENs. Fourth- and fifth-generation PAMAM dendrimers can be considered ‘hybrids’ in the sense that both DENs as well as DSNs can form^14,20^. By incorporating dendrimers into the micelles, the unique encapsulation property of dendrimers can be used to “load” complex coacervate core micelles, as we recently showed by combining block copolymers together with DENs^21,22^, forming well-defined dendrimicelles that carried ~20–30 nanoparticles/dendrimicelle depending on the generation used. These nanoparticle-containing dendrimicelles self-organized into distinct disk-like monolayer superstructures inside ~0.1 µm thick biconcave ice films formed during cryoTEM sample preparation^21^.

The dendrimicelles arranged following the thickness of the thin, biconcave-shaped, ice film, related to the increased size-sorting to an increased dendrimicelle polydispersity caused by a decreased micelle stability, corroborating with previous work by Wang *et al*. on carboxylic acid-terminated PAMAM dendrimers that showed a generation-dependent dendrimicelle stability^13^. To better understand the properties of dendrimicelles formed from dendrimer generations <6, we reasoned that gold particles embedded in the micellar core could reveal details on composition shape, and stability of the micellar subcomponents with unprecedented detail. The decreased stability of lower generation-based dendrimicelles is expected to lead to an increased dendrimicelle polydispersity at off-stoichiometric charge mixing fractions. In turn, the increased polydispersity is expected to lead to a more pronounced size sorting of dendrimicelle inside dendrimicelle superstructures formed during cryoTEM sample preparation^21^.

**Figure 1:**
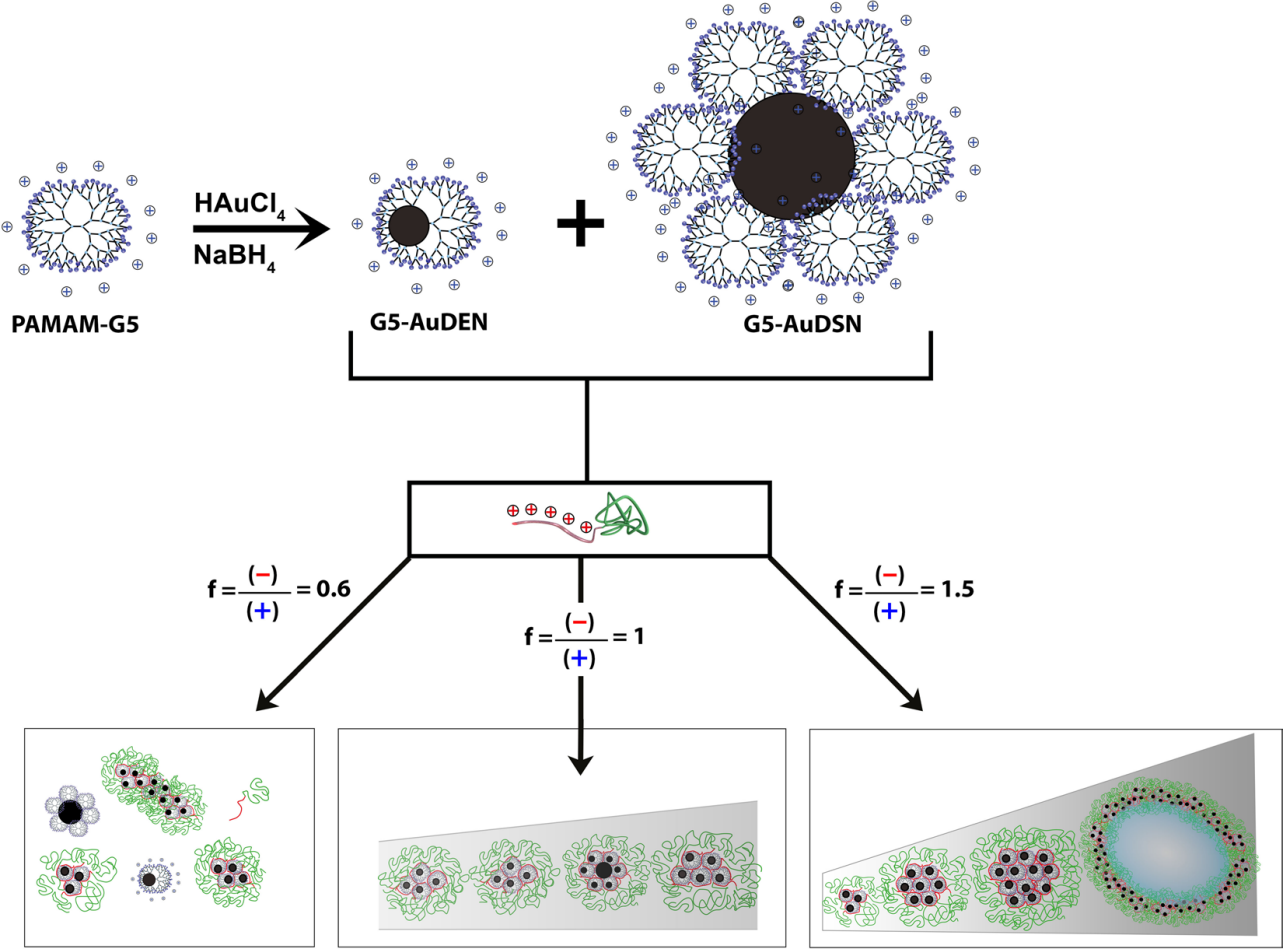
Preparation of dendrimicelles from amine-terminated fifth generation PAMAM dendrimers. Complexation and reduction of Au^3+^ ions inside fifth-generation PAMAM results in DENs as well as DSNs. Addition of a negative-neutral block copolymer, pMAA_64_pEO_885_ results in formation of dendrimicelles, depending on the charge fraction f, where f = (negative charge from the block copolymers)/(positive charge from the dendrimers). Charge stoichiometric mixing (f = 1) yields well-defined dendrimicelles. Using excess block copolymer to dendrimer (f = 1.5) results in coacervate structures with an increased polydispersity: dendrimicelles as well as much bigger nanostructures, such as complex coacervate vesicles.

We here present dendrimicelles formed from generation five-based PAMAM dendrimers at various charge stoichiometries (see Fig. 1), and analyze the formed complex coacervate superstructures using cryoTEM and Dynamic Light Scattering techniques. The synchronous formation of DSNs as well as DENs is shown, which co-assembled into well-defined dendrimicelles at charge-stoichiometry. Using off-stoichiometric mixing conditions yields samples with a high polydispersity, in which an amplified size-sorting inside the formed superstructures is observed and in which, next to dendrimicelles, additional nanoaggregates form.

**Figure 2:**
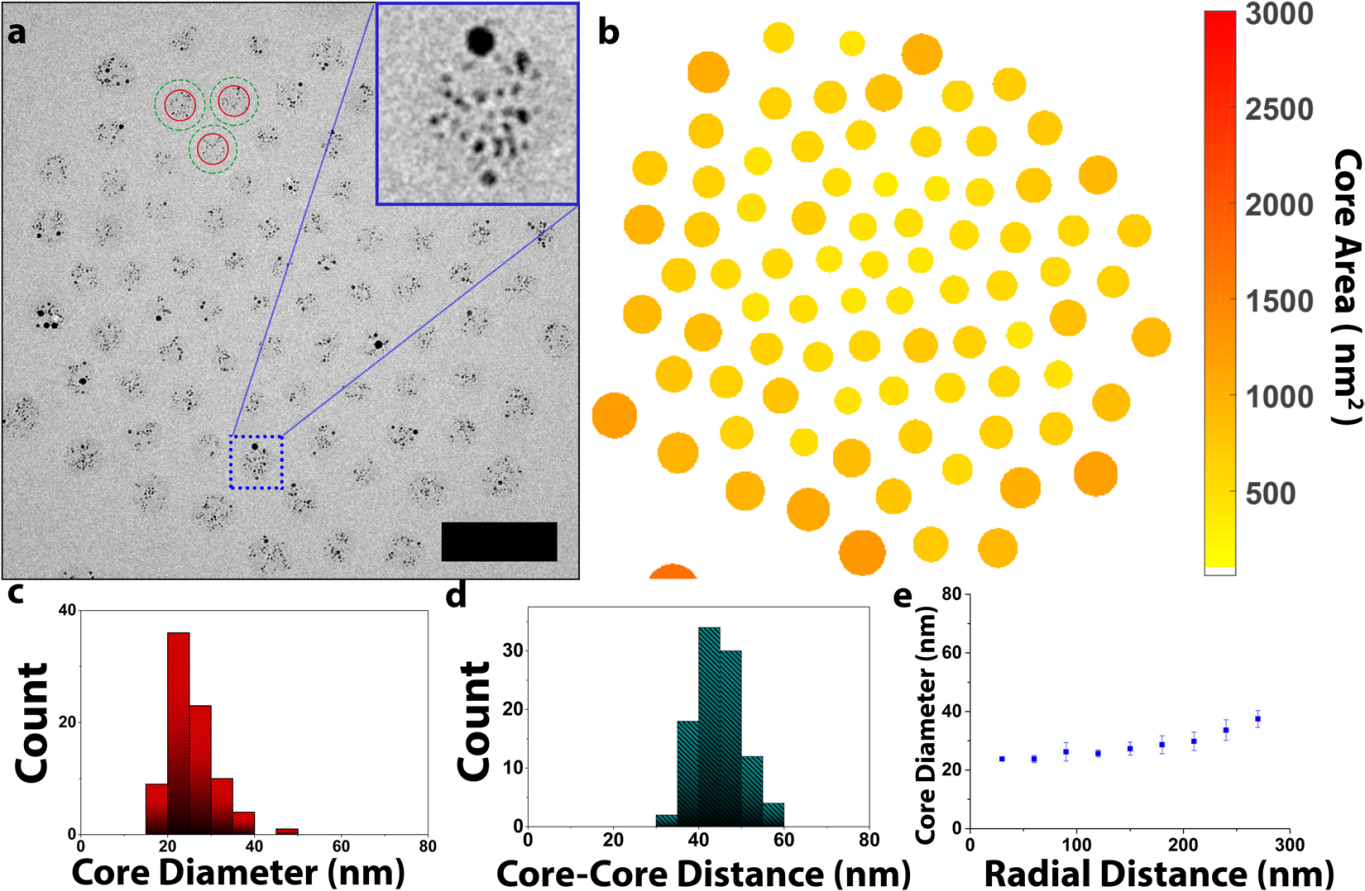
PAMAM generation 5-based dendrimicelles made at charge-stoichiometry. **(a)** cryoTEM micrograph of the dendrimicelle superstructure, with the (40 × 40 nm) inset showing a single dendrimicelle; here, the core clearly reveals smaller gold particles (DENs) as well as ~6 nanometer big particles (DSNs) The red, solid circles in this figure indicate the dendrimicelle core, as identified by the embedded nanoparticles. The green, dotted circle indicates the total dendrimicelle size, as determined from DLS. Scale bar is 100 nm. **(b)** Heat map plot of dendrimicelle superstructure, with the individual dendrimicelles color-coded according to the core area. **(c)** The average dendrimicelle core diameter is 26 ± 6 nm. (**d**) The average dendrimicelle diameter, as determined from measuring dendrimicelle core-core distances is 45 ± 5 nm. **(e)** Plotting the micelle core area versus the radial distance to the center of the dendrimicelle superstructure shows a slight size-sorting of the dendrimicelles over the superstructure.

Au_64_DENs were synthesized inside fifth-generation poly(amidoamine) (PAMAM) dendrimers by complexation and subsequent reduction of Au^3+^ inside the PAMAM dendrimer^14^. TEM analysis showed the formed nanoparticles to be 2.3 ± 0.8 nm (Figs S1, S2), bigger than the expected size of Au_64_DENs^23^. Fitting the obtained size distribution with 2 Gaussian distributions indicates the presence of two populations of nanoparticles, one centered around ~1.6 nm, and one centered at ~2.5 nm. The observed polydispersity, and the observation of two nanoparticle populations in Figs S1, 2, suggests the formation of DSNs as well as DENs. We do note here that the average nanoparticle size of the DENs population (1.6 nm) is slightly higher than expected (1.3 nm), which —more often observed for DENs— could be attributed to steric constraints in the dendrimer core^19^. Alternatively, the DENs populations could actually exist as a mix of real DENs, i.e. one-on-one dendrimer-nanoparticles, with smaller DSNs, i.e. one nanoparticle with two or three dendrimers. Following the strategy that we reported before^22^, we then mixed the AuDENs with an anionic–neutral block copolymer, consisting of a 64-subunit polymethacrylic acid and a 885-subunit polyethyleneoxide block, pMAA_64_pEO_885_. Coacervation of the cationic dendrimer with the anionic-neutral block copolymer resulted in the formation of a complex coacervate core micelle, as indicated by Dynamic Light Scattering, DLS. Charge-stoichiometry was found at a ~1:1 combination of positive charge (dendrimer) to negative charge (block copolymer) (See Fig. S3), and the CMC of the formed dendrimicelles was determined to be ~1 mg.L^−1^ (Fig. S4). Cryo-Transmission Electron Microscopy, cryoTEM, confirmed that the structures observed with DLS at charge-stoichiometry are indeed well-defined dendrimicelles (Figs 2 & S5–6), and revealed the dendrimicelle core diameter, as indicated by the gold nanoparticles, to be 26 ± 6 nm (Fig. 2c). The dendrimicelle diameter, as determined by measuring core-core distances, is 45 ± 5 nm (Fig. 2d), in good agreement with the size as determined by DLS (See Fig. S5).

The inset in the cryoTEM image in Fig. 2, however, illustrates that next to the many ~1–2 nm-sized nanoparticles, also a 6 nm gold particle is encapsulated in the dendrimicelle core. Since the diameter of a fifth-generation PAMAM dendrimer is about 5 nm, such a 6 nm gold nanoparticle does not fit inside a single dendrimer, and therefore has to be considered a DSN rather than a DEN. Interestingly, the data discussed above infer that both DSNs as well as DENs can be encapsulated inside dendrimicelles. The DSNs consist of a gold nanoparticle with multiple dendrimers passivating the surface, but still a great part of the dendrimer, including its charged groups, is exposed to the solution and hence are available for charge interactions to allow C3M formation. The unknown number of dendrimers surrounding a DSN, as well as the large number of AuDENs inside every dendrimicelle hinder accurate quantification of the micelle aggregation numbers by simply counting the number of nanoparticles per dendrimicelle. Counting the number of nanoparticles per dendrimicelles showed 27 ± 11 nanoparticles per dendrimicelle (Fig. S7).

Assuming AuNPs >2 nm to be DSNs indicates that f~0.6 of the AuNPs observed in Figs S1, S2 are DSNs. The average nanoparticle size of a DSN is ~2.8 nm, suggesting that ~7 dendrimers surround a DSN. Correcting the observed number of nanoparticles per dendrimicelle (e.g., both DENs and DSNs) for the number of dendrimers surrounding a DSN suggests an average of 1.2*10^2^ dendrimers per dendrimicelle. Assuming that 50% of the dendrimer terminal amines of a DSN is available for coacervating, and the 1:1 association of (available) positive and negative charges, the average micelle molecular weight can be guesstimated to be ~11 MDa (See Supporting Info for calculations and assumptions made). Extending on these calculations, the packing fraction of the dendrimers in the dendrimicelle core (not taking into account the charged part of the block copolymer) is ~0.9, high yet in line with the packing fractions found previously for generation 7- through 9-based dendrimicelles^21^. Although these calculations remain rather approximate, and we cannot exclude the presence of empty dendrimers in solution after nanoparticle formation, the calculated dendrimer packing fraction inside the dendrimicelle infers that only a few empty dendrimers could be present at most.

To investigate the effect of empty dendrimers on the formation of dendrimicelles, we prepared a 1:1 mixture (based on total number of dendrimers) of ‘empty’ and nanoparticle-engaged dendrimers, followed by charge-stoichiometric addition of block copolymer. The formed dendrimicelles (See Fig. S6) were almost identical to those shown in Fig. 2, except for the number of nanoparticles observed per dendrimicelle (12 ± 5 *versus* 27 ± 11). This confirms that empty dendrimers co-encapsulate with nanoparticle-containing dendrimers, in line with previous results^22^.

Analysis of the dendrimicelle superstructure shown in Fig. 2a using image segmentation, and subsequent color-coding of the determined dendrimicelle core area, suggests a slight size-sorting present in the dendrimicelle superstructure (See Fig. 2b). We quantified the size sorting by plotting the average dendrimicelle core diameter versus the radial distance to the center of the dendrimicelle superstructure, as shown in Fig. 2e. This figure indicates that dendrimicelles located at a radial distance of ~60 nm from the center have an average core diameter of ~24 nm, whereas the dendrimicelles located at a radial distance of ~240 nm have a core diameter of ~34 nm, endorsing the size-sorting of dendrimicelles inside the biconcave-shaped ice layer.

**Figure 3:**
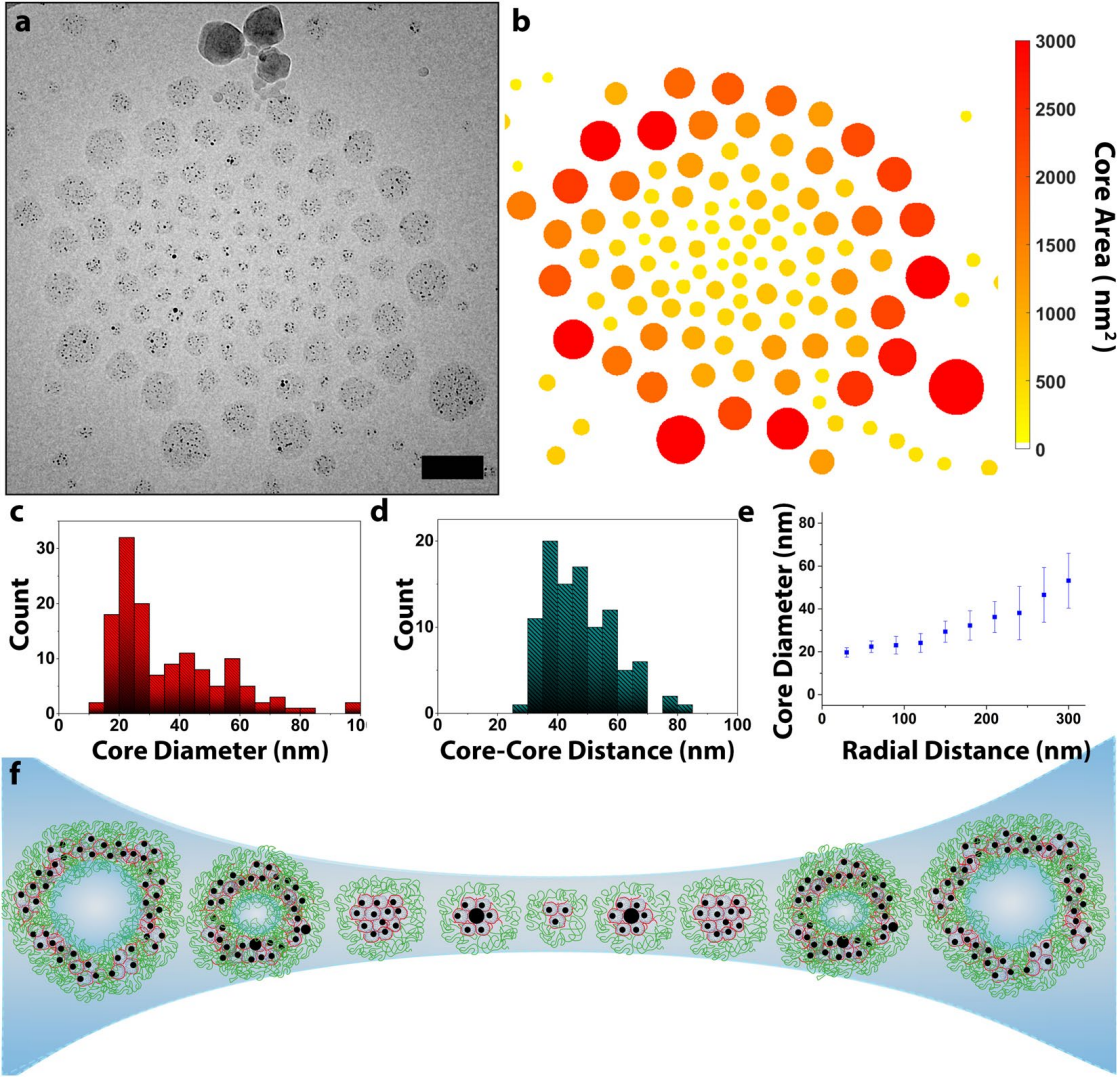
Extreme size-sorting of dendrimicelles made under off-stoichiometric conditions. **(a)** CryoTEM micrograph of the formed dendrimicelle superstructure. **(b)** Heat map plot of dendrimicelle superstructure, with the individual dendrimicelles color-coded according to the core area as determined from the cryoTEM micrograph in **(a)**, emphasizing the size-sorting present. **(c)** The average micelle core diameter is 36 ± 18 nm. **(d)** The average micelle size, as determined from the micelle core-core distances is 48 ± 12 nm. **(e)** Plotting the micelle core area versus the radial distance to the center of the dendrimicelle superstructure confirms the size-sorting of the dendrimicelles. **(f)** Schematic illustration of the amplified thin film-templated size sorting. Scale bar is 100 nm.

By forming generation five-based dendrimicelles using an excess of one of the building blocks, we attempted to increase the polydispersity. Initially, we prepared a dendrimicelle sample at a charge fraction f = 0.6 (i.e. 40% less block copolymer than needed to achieve charge-stoichiometry). At this mixing fraction, however, the scattered light intensity is considerably lower, indicating that despite some dendrimicelles form (Fig. S3, 8), they are likely ill-defined. Indeed, Fig. S9 depicts a representative cryoTEM micrograph of this sample, corroborating that the structures observed with DLS are ill-defined and non-spherical. Next, we prepared a generation five-based sample using excess block copolymer to dendrimer (i.e., at charge fraction f = 1.5). Analysis of this sample using DLS indicated that dendrimicelles with a hydrodynamic diameter of ~50 nm formed (Fig. S10). CryoTEM micrographs of these dendrimicelles (Figs 3 and S11) show that the polydispersity of the sample increased with respect to the charge-stoichiometrically prepared sample. Alternatively, one might hypothesize that all micelles present on the grid are of similar size, and just squeezed by the forces imposed on the dendrimicelles in the biconcave interface obtained during cryoTEM sample preparation. This would then affect the apparent shape of the dendrimicelles present, flattening them and resulting in discoid forms. However, assuming equally sized dendrimicelles to be present over the whole TEM grid-hole area, then, flattening of the dendrimicelles in the center of the biconcave film (where it is thinnest)^21^ would cause an increased ‘squeezing’ of the micelles, resulting in a concomitant inversed size-sorting pattern, i.e. larger appearing micelles in the center and smaller ones toward the outside; however, this is clearly not what is observed. The average dendrimicelle core diameter, as determined from the cryoTEM images, was 36 ± 18 nm for the f = 1.5 sample, compared to 26 ± 6 nm for the f = 1 sample, demonstrating that both the average size, as well as the standard deviation are increased in the f = 1.5 sample.

Analysis of the cryoTEM micrographs showed the largest nano-assemblies in Fig. 3 have a “core” diameter of ~90 nm. As the methacrylic acid block of the block copolymer we use here, pMAA_64_pEO_885_, has a contour length of well under 20 nm^24^, and the “core” radius is about 50 nm, the charged block of the block copolymer is not long enough to fill and form the core of such a size complex coacervate core micelle. Furthermore, the core size distribution plot, as shown in Fig. 3c, indicates there are two different populations present. This supports our claim that the in Fig. 3 observed nanostructures with a ‘core’ >40 nm are some other form of nano-assembly rather than dendrimicelles. The presence of a second type of nano-assemblies besides dendrimicelles would also explain the two populations that appear to be present in Fig. 3c. Therefore, these large structures observed are likely not dendrimicelles, but rather vesicles, as schematically drawn in Fig. 1^25^. Plotting the volume-per-nanoparticle ratio (Fig. S12) showed the observed structures with apparent core diameters >30 nm to be significantly different in volume-per-nanoparticle ratio (p < 0.05) from the dendrimicelles population, supporting the hypothesis that these are different, i.e. vesicle-like, structures. The question might arise whether these larger structures are formed already in the micelle preparation step, or during cryoTEM preparation step where the biconcave water layer can act as template inducing forces on the complex coacervate structures. We observe for dendrimicelle samples based on 5^th^ generation PAMAM, that over the course of a month DLS shows an increase of the average size of the assemblies from 50 to 80 nm, and hence the vesicle –like structures form, also, independently of the cryoTEM preparation steps.

The cryoTEM micrograph (Fig. 3a) shows that the increased polydispersity in this sample translated into more distinctive size-sorting in the complex coacervate superstructures. This effect is visually emphasized when dendrimicelle core-size based color-coding is applied (See Fig. 3b). We quantified the size sorting by plotting the average core diameters versus the radial distance to the center of the superstructure (Fig. 3e). Interestingly, the slope of the radial distribution plot also suggests the presence of two separate nanoaggregate populations. Dendrimicelles in the center of the superstructure have a core diameter of about 20 nm, whereas the nano-assemblies residing at the edge of the superstructure reach ‘core’ diameters up to ~100 nm. In this plot, a doubling of the core diameter occurs over a radial distance of ~250 nm, demonstrating that here the increased polydispersity leads to amplified size-sorting in the formed superstructures. It is important to notice that different generations of dendrimers yield dendrimicelles with different properties. For the generation seven (and higher)-based dendrimicelles that we reported on before^21^, we observed a doubling of the core diameter over a radial distance of ~500 nm (from 20 to 40 nanometer), demonstrating that the increased polydispersity leads to amplified size-sorting in the formed superstructures. The minimum and maximum sizes observed are consistent with dendrimicelles. On the other hand, the generation-5 based aggregates presented here, show relatively extreme size differences, up to five-fold bigger over a radial distance of ~500 nm (from 20 to 100 nanometer).

We demonstrate here how gold nanoparticles, synthesized inside fifth-generation PAMAM dendrimers, form DENs as well as DSNs, which both are embedded inside well-defined dendrimicelles upon charge-stoichiometric mixing with an oppositely-charged block copolymer. Ill-defined nanostructures form in the case of excess dendrimer to block copolymer, whereas excess block copolymer to dendrimer provides more explicitly defined nanostructures with an increased size and polydispersity. This increased polydispersity translates into an amplified size sorting inside formed superstructures. The nanoparticle-loaded dendrimicelles are a powerful tool to investigate the formed structures with cryoTEM, even for systems in which the subcomponents consist of mixtures of different populations of dendrimer-particles ratios like for the fifth generation PAMAM DENs. Besides well-defined dendrimicelles, also nanostructures were observed that are too large to be complex coacervate core micelles, but more likely vesicles. The incorporation of both DENs and DSNs inside the complex coacervate core dendrimicelles is, for example, of interest for catalytic applications, as the catalytic activity of nanoparticles is dependent on, among others, the nanoparticle size^26,27^. Both the catalytic activity inside dendrimicelles as well as the formed vesicles are currently investigated.

## Materials and Methods

### Materials

Amine-terminated polyamidoamine (PAMAM) dendrimers, (3-(N-morpholino)-propanesulfonic acid) (MOPS), NaBH_4_, 1 M NaOH and 1 M HCl solutions were obtained from Sigma Aldrich. pMAA_64_-b-PEO_885_ (Mw/Mn = 1.15) was obtained from Polymer Sources Inc., Canada and used as 5 mM solution based on carboxylic acid content. HAuCl_4_.3H_2_O was obtained from TCI.

### Dendrimer encapsulated nanoparticles

G5-Au_64_DENs were made following established protocols^14^. Shortly, 50 µL (69 nmol) of 5 wt% PAMAM G5-NH_2_ in methanol was transferred to a 5 mL vial and the methanol was evaporated under reduced pressure. Next, 2 mL of water was added to dissolve the PAMAM and the pH was adjusted to 3 using 1 M HCl, after which 64 molar equivalents of Au^3+^ to PAMAM were added. This solution was stirred for 20 minutes, after which 44 µL of a 1 M solution of NaBH_4_ in 0.3 M NaOH (10 molar equivalents to Au^3+^) were added. This resulted in the reduction of Au^3+^ to AuDENs, indicated by the change from colorless to a dark brown solution within seconds after addition. After reduction, the pH was set to 7 using HCl and the DENs were stored at 4 °C.

### Dendrimer-encapsulated-nanoparticle micelles

To obtain dendrimicelles under charge stoichiometric conditions, 20 µL of 2.9 mM dendrimer solution (charge concentration, corresponding to 59 nmol positive charge based on surface groups) was dissolved in 149 µL water and 20 µL of 0.2 M MOPS buffer at pH 7 was added. Then, 11 µL pMAA_64_-b-PEO_885_ (55 nmol based on -COOH) was added under sonication, and the sample was sonicated for 2 minutes total. Dendrimicelles at off-stoichiometric charge mixing fractions were made by adjusting the amount of pMAA_64_PEO_885_ added, keeping the total volume constant at 200 µL.

### Methods

Dynamic Light Scattering (DLS) was done on a Malvern Zetasizer Nano S equipped with a laser operating at 633 nm. Sample grids for electron microscopy were obtained from Electron Microscopy Sciences (EMS, Hatfield, PA, USA) and were rendered hydrophilic using a plasma cleaning setup (~15 s at 10^−1^ Torr). CryoTEM samples were cast on Quantifoil R2/2 grids. After blotting, samples were plunged into liquid ethane using a Vitrobot system (FEI Company). Samples were then imaged at ~90–100 K in a JEOL 2100 TEM operating at 200 kV or JEOL 1400Plus TEM operating at 120 kV. UV-Vis absorbance was evaluated to observe SPR shifts upon formation of micelle cores and concomitant approximation of the distance between gold nanoparticles; however, this was not evident (Fig. S13).

### Image analysis

CryoTEM images were analyzed using ImageJ and custom Matlab micelle tracking script, as reported before^21^. The average core-core distance was calculated by measuring 100 randomly selected neighboring dendrimicelles. Dendrimicelle core areas were determined from measuring the area of the circle surrounding core, as indicated by the AuNPs embedded within.

## Electronic supplementary material

Supplementary Information
