# Supplementary material for: Nanoparticles reveal Extreme Size-Sorting and Morphologies in Complex Coacervate Superstructures
Source: Sci Rep. 2018 Sep 14;8:13820. doi: 10.1038/s41598-018-32240-5 (PMC6138629; doi:10.1038/s41598-018-32240-5)
Supplement: Supplementary file 1 — Supplementary Information [file 41598_2018_32240_MOESM1_ESM.pdf]

Supporting Information

Nanoparticles reveal Extreme Size-Sorting and Morphologies in Complex Coacervate Superstructures

Jan Bart ten Hove[1,2], Matthias N. van Oosterom[2], Fijs W.B. van Leeuwen[1,2], Aldrik H. Velders[1,2]*

1) Laboratory of BioNanoTechnology, Wageningen University, Bornse Weilanden 9, Wageningen, The Netherlands

2) Interventional Molecular Imaging Laboratory, Leiden University Medical Centre, Leiden, The Netherlands

Guestimation of the dendrimicelle molecular weight.

The polydispersity of the nanoparticles, as well as the presence of both dendrimer-encapsulated (DENs) and –stabilized nanoparticles (DSNs) hinders the accurate calculation of the dendrimicelle molecular weight. However, by making some assumptions and estimations we can make a guesstimation of the dendrimicelle molecular weight. Hereto, first, we assume the dendrimicelles to be spherical; and to calculate the average micelle molecular weight, the ratio between DSNs:DENs should be known. Based on Figure S1/S2, nanoparticles >2 nm are assumed to be DSNs. This indicates that the fraction of nanoparticles in the form of DSNs is ~0.6, with an average DSN diameter of 2.8 nm. Assuming the dendrimer radius to be 2.5 nm and its packing area to be $\pi*r^2$, the DSNs to be spherical, with a surface area of $4\pi*(rDSN,avg+rPAMAM)^2$ and the packing density of dendrimers per DSN of 0.7, this infers an average of 7 dendrimers per DSN.

CryoTEM indicated an average of 27 nanoparticles per dendrimicelle, of which ~61% are DSN, and ~39% are DENs. Correcting the number of counted nanoparticles by the number of dendrimers/DSN suggests the number of dendrimers engaged with a nanoparticle in a micelle to be ~$1.2*10^2$Since dendrimers in DSNs only expose part of their terminal amines to the solution, we assume 50% the dendrimer terminal amines to play a role in the complex coacervate core of the micelle. The other 50% charge is assumed to be inaccessible by the block copolymer or to interact with the gold nanoparticle. Taking a 1:1 interaction of available dendrimer charge (of the DSNs and DENs) with the block copolymer suggests the presence of ~$1.3*10^2$ block copolymers per dendrimicelle. Assuming all gold ions have been converted into gold particles, either DENs or DSNs, and assuming the fraction of non-engaged is small, the average number of gold atoms per dendrimer is presumed to be 64. Finally, the micelle molecular weight is then calculated from the molecular weight of the polymer (~45 kDa), the dendrimer (~30 kDa) and of the average gold nanoparticle per ideal Au_{64} DEN (13 kDa), yielding an average dendrimicelle molecular weight of ~11 MDa.

Guestimation of dendrimicelle volume per dendrimer

The core diameter of PAMAM generation five-based dendrimicelles was determined to be ~26 nm (Figure 2c), corresponding to a core volume of ~$9*10^3$ nm^3. Division of the dendrimicelle core volume by the actual number of dendrimers per dendrimicelle suggests ~$8*10^1$ nm^3 per dendrimer present in the complex coacervate core. Note: This volume is not the volume that a single dendrimer occupies, but rather the sum of the contributions of (dendrimer + charged block copolymer + water). As the volume of a single fifth generation PAMAM dendrimer is ~$7*10^1$ nm^3, this indicates that about 88% of the dendrimicelle core consists of dendrimer, in line with previous findings on dendrimicelles with higher eneration PAMAMs. [21]

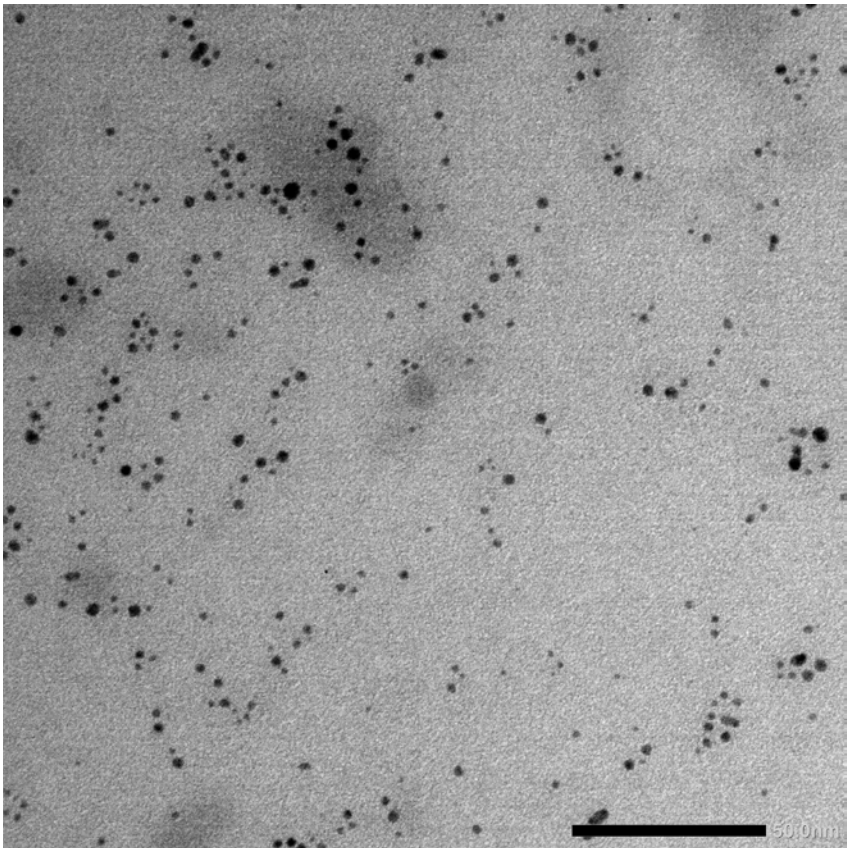

Figure S1. TEM micrograph of PAMAM G5-Au_{64} dendrimer-encapsulated nanoparticles. The many ~1 nm-sized nanoparticles are dendrimer-encapsulated nanoparticles, and the few large nanoparticles are likely dendrimer-stabilized nanoparticles (DSNs). The scale bar is 50 nm.

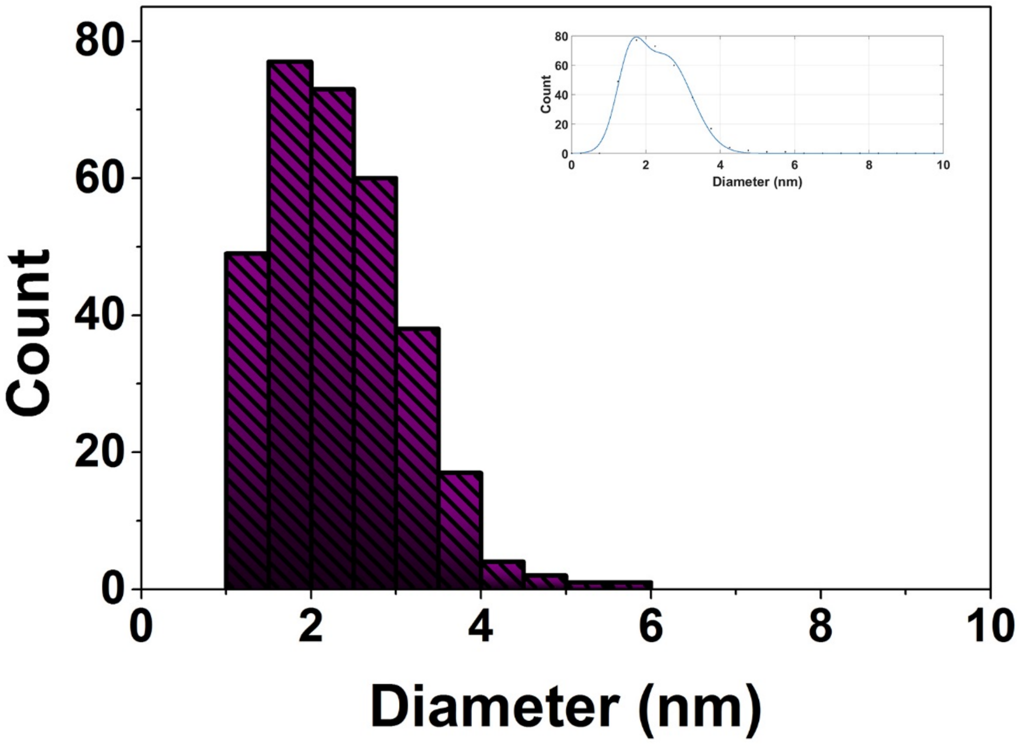

Figure S2. Histogram showing the mean diameter of the obtained G5-Au_{64} DENs as shown in Figure S1. The average diameter was determined to be 2.3 ± 0.8 nm, indicating the presence of both DENs as well as DSNs. Inset: Fitting the obtained size distribution with 2 Gaussian distributions (R^2 > 0.99) indicated the presence of two populations of nanoparticles, one centered around ~1.6 nm, and one centered at ~2.5 nm. 322 nanoparticles were analyzed for the size histogram.

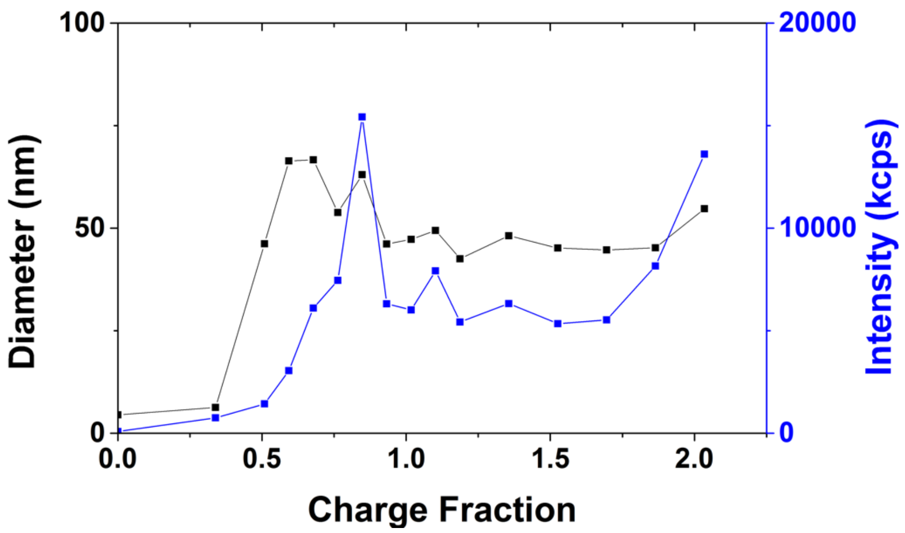

Figure S3. DLS charge titration graph of PAMAM G5-NH_2 with $pMAA_{64}PEO_{885}$, showing the number-averaged dendrimicelle diameter in blue and the normalized scattered light intensity in black against the charge fraction. The amount of positive charge (dendrimer-NH_2) was kept constant at 59 nmoles, and the amount of negative block polymer was varied, while keeping total volume constant. The charge fraction was calculated as the ratio of (COOH/NH_2).

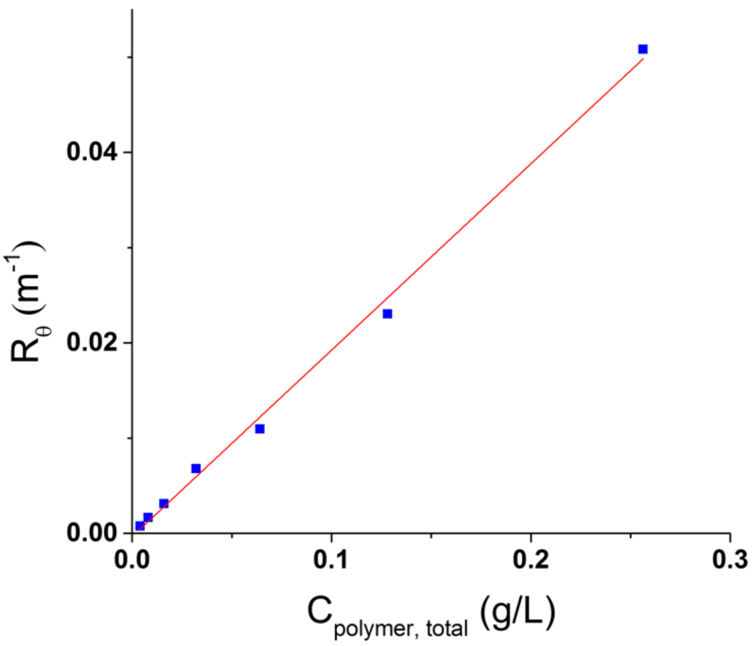

Figure S4. CMC determination of dendrimicelles made from fifth generation poly(amidoamine) dendrimers. The intensity (as the Rayleigh ratio) is plotted versus the total concentration of polymer (dendrimer + block copolymer). Fitting the data points with a linear fitting formula (R^2=0.996), and extrapolating to zero intensity gives a CMC of ~1 mg.L^{-1}.

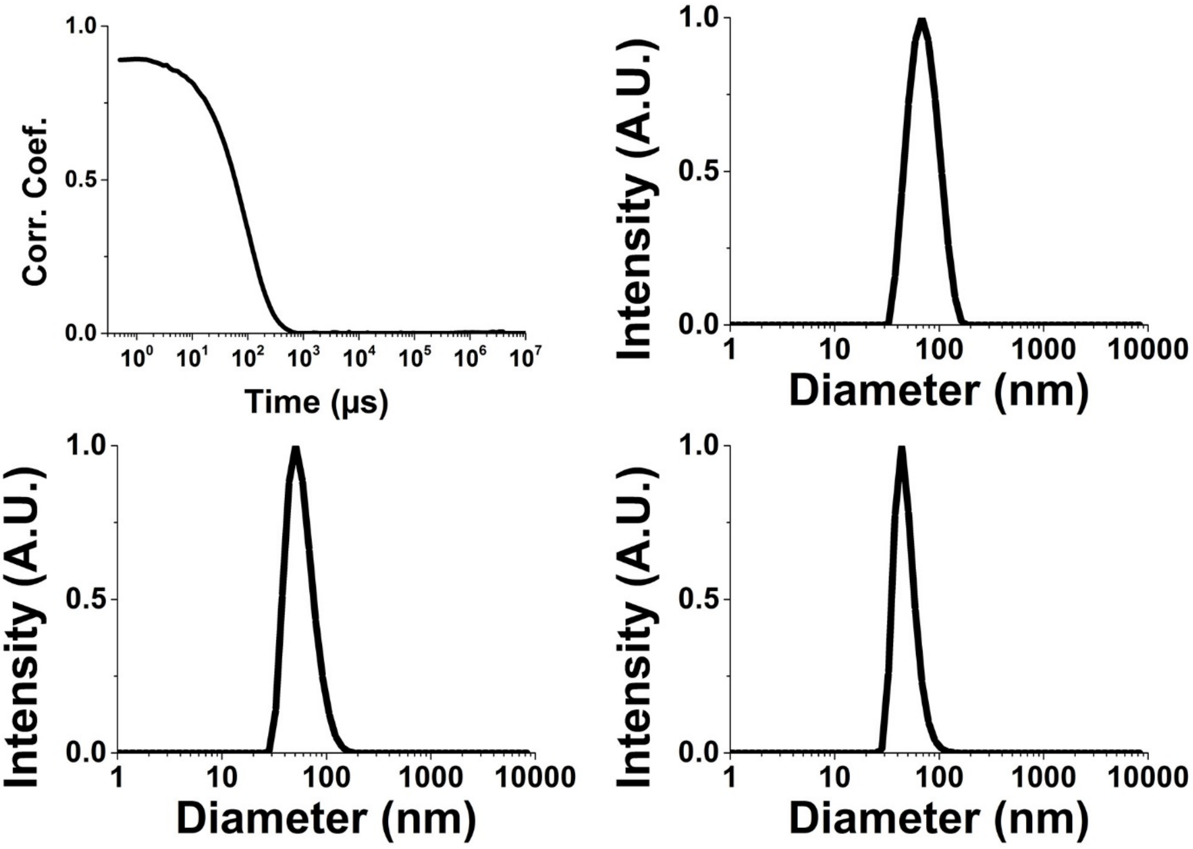

Figure S5. DLS characterization of dendrimicelles made at charge-stoichiometry (f=1). Top-left: DLS autocorrelation plot. Top-right: Intensity-weighed size plot, indicating an average size of ~73 nm. Bottom-left: Volume-weighed size plot, indicating an average size of ~58 nm. Bottom-right: The number-weighed DLS size plot indicates an average micelle hydrodynamic diameter of 48 nm.

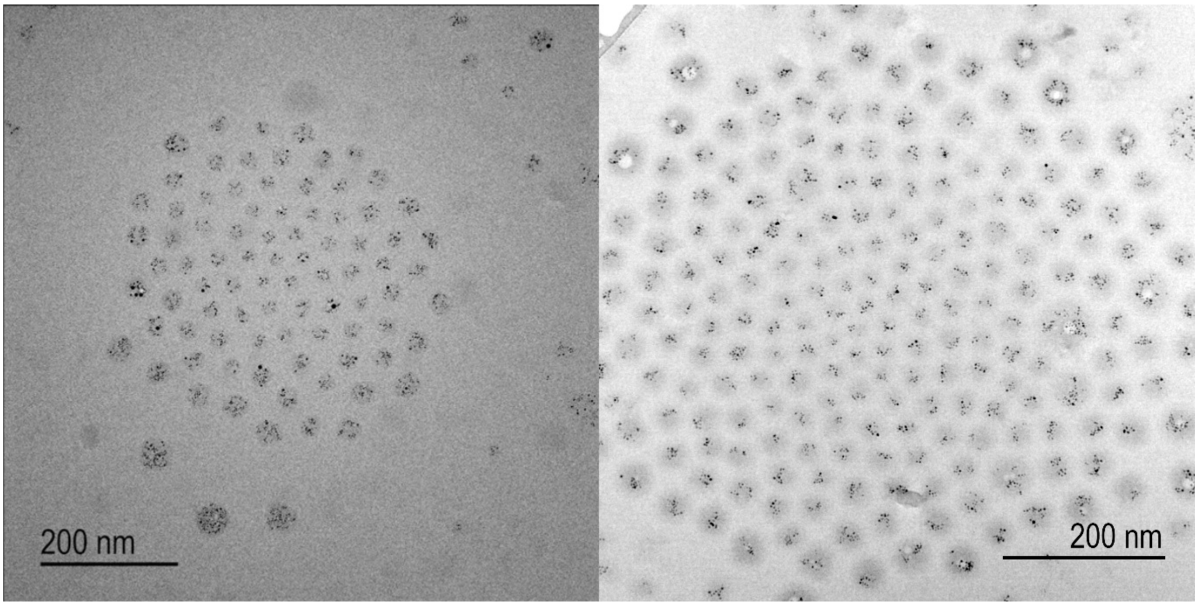

Figure S6. cryoTEM micrograph of PAMAM-G5 dendrimicelles prepared at charge-stoichiometry. Micelles in the left image were made from the G5-DENs/DSNs sample, whereas those in the right image were made from a 1:1 mix of G5-DENs/DSNs:G5-NH_2. DLS indicated a micelle hydrodynamic diameter of ~50 nm for both samples. The micelles in the left image contain 27 ± 11 nanoparticles and have a micelle core area of 26 ± 6 nm; those in the right image contain 12 ± 5 nanoparticles per micelle, have an average dendrimicelle core-core distance is 46 ± 8 nm, and have a micelle core area of 26 ± 7 nm.

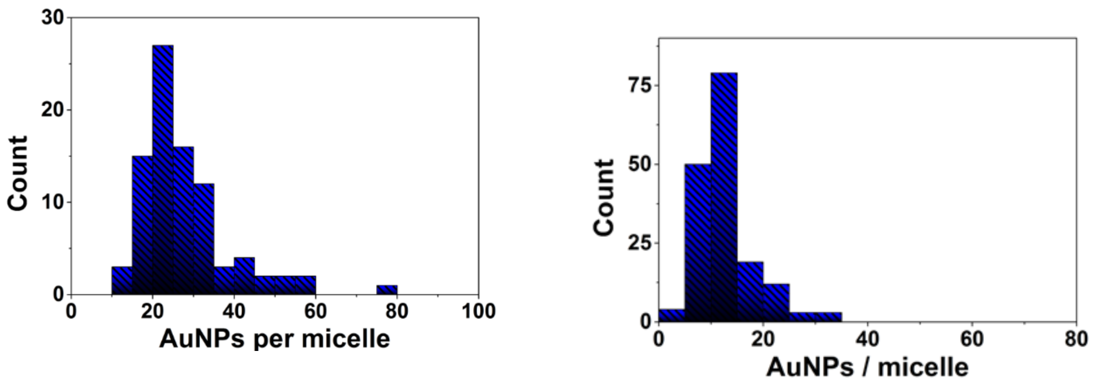

Figure S7. Size histograms showing: (left) the number of AuNPs per dendrimicelle for fifth-generation based dendrimicelles made at charge-stoichiometry from the G5-DENs/DSNs sample. The average counted number of gold nanoparticles per dendrimicelles is 27 ± 11. We do note that here the large number of nanoparticles per micelle, together with the presence of DSNs among the DENs hinders the accurate determination of the dendrimicelle aggregation numbers. (right) The number of AuNPs per dendrimicelle for fifth-generation based dendrimicelles made at charge-stoichiometry from a 1:1 mix of G5-DENs/DSNs with empty dendrimers. The average counted number of gold nanoparticles per dendrimicelles is 12 ± 5.

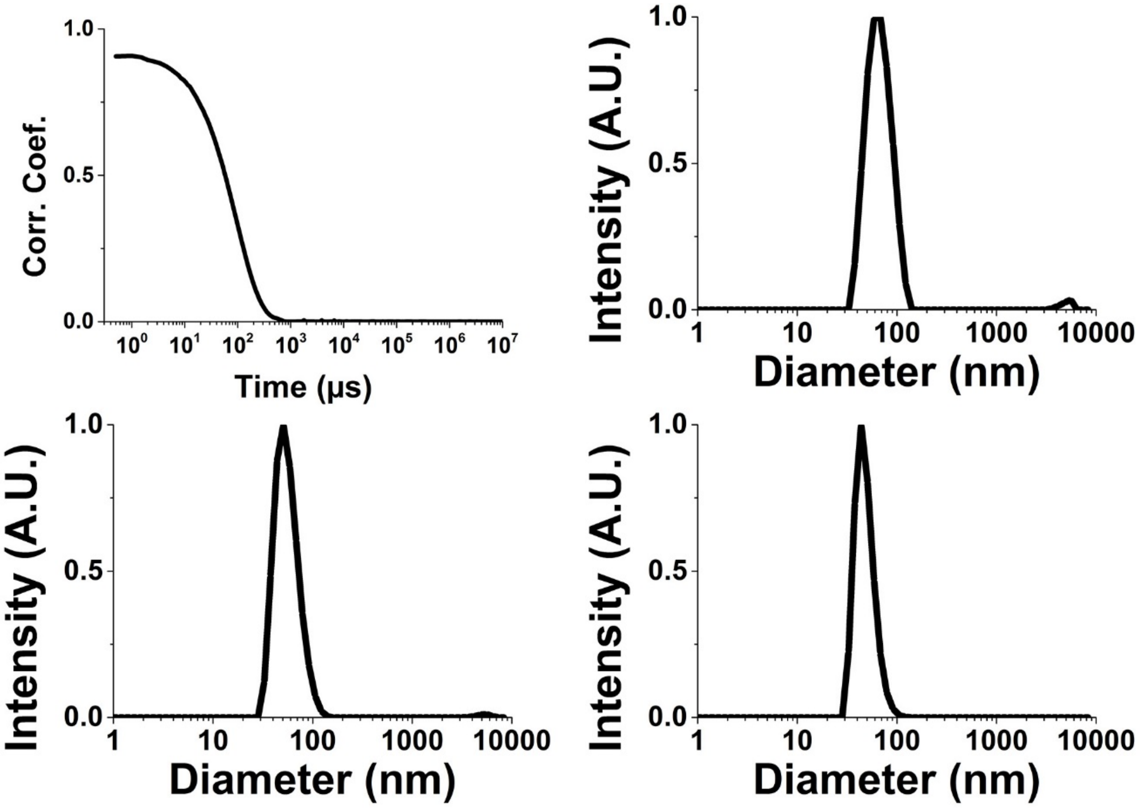

Figure S8. DLS characterization of dendrimicelles made under off-stoichiometric charge mixing conditions (f=0.6). Top-left: DLS autocorrelation plot. Top-right: Intensity-weighed size plot, indicating an average size of ~138 nm. Bottom-left: Volume-weighed size plot, indicating an average size of ~90 nm. Bottom-right: The number-weighed DLS size plot indicates an average micelle hydrodynamic diameter of 48 nm.

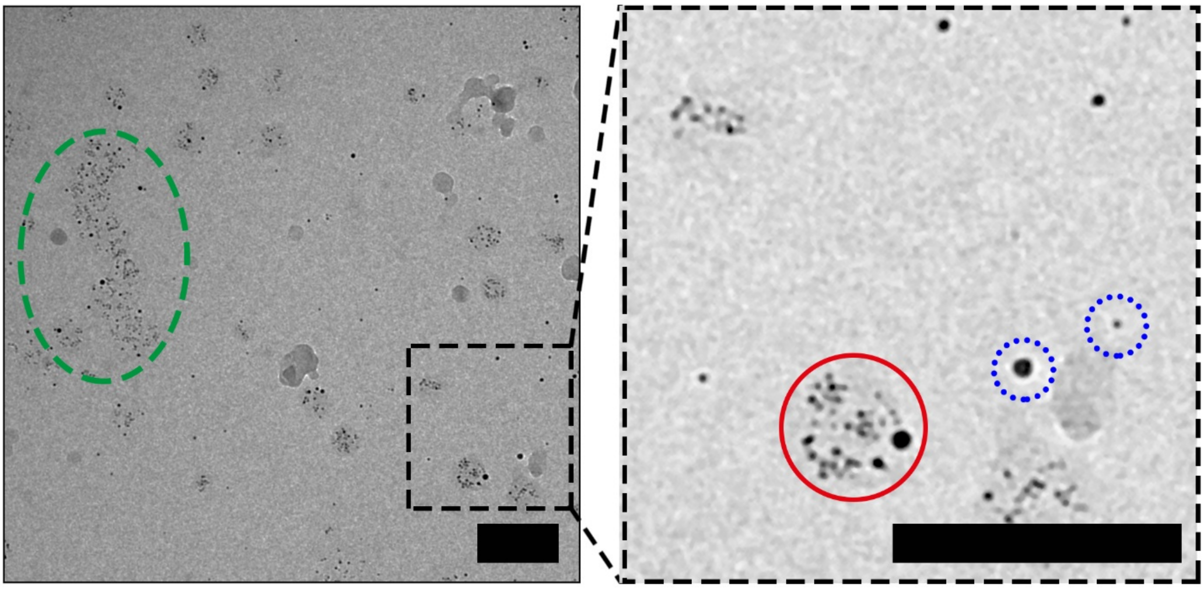

Figure S9. Representative cryoTEM micrograph of generation five-based dendrimicelles prepared at a charge mixing fraction f=0.6, corresponding to excess an excess of dendrimer to block copolymer. Clearly, the solution contains a mixture of large aggregates (dashed, green, oval), dendrimicelles (solid, red, circles) and individual nanoparticles (DENs and DSNs, indicated with the dotted, blue, circles). The scale bars represent 100 nm.

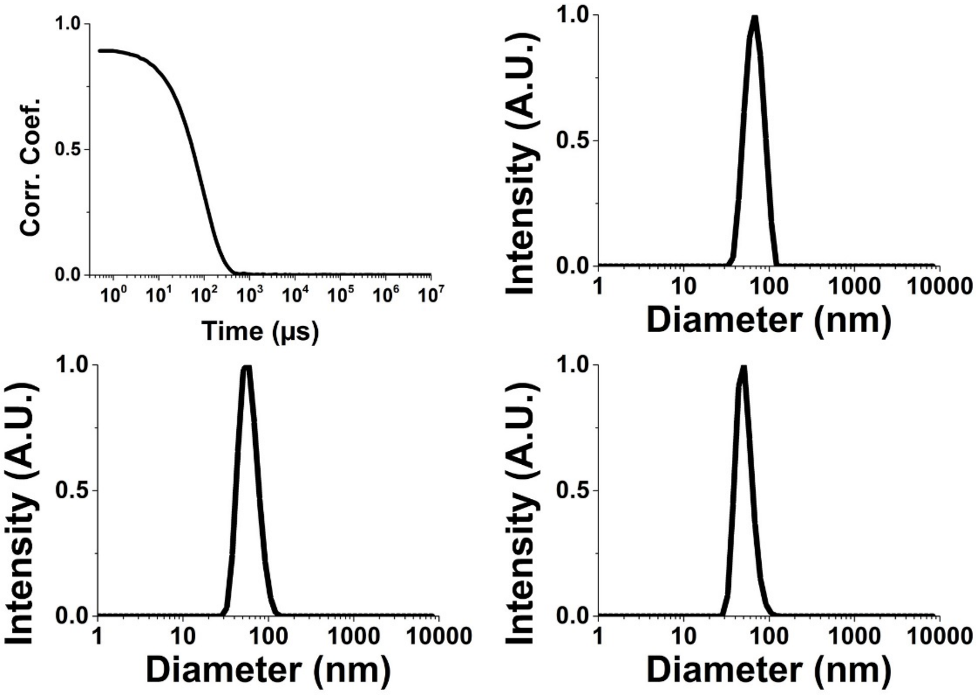

Figure S10. DLS characterization of dendrimicelles made under off-stoichiometric mixing conditions (f=1.5). Top-left: DLS autocorrelation plot. Top-right: Intensity-weighed size plot, indicating an average size of ~68 nm. Bottom-left: Volume-weighed size plot, indicating an average size of ~60 nm. Bottom-right: The number-weighed DLS size plot indicates an average micelle hydrodynamic diameter of 52 nm.

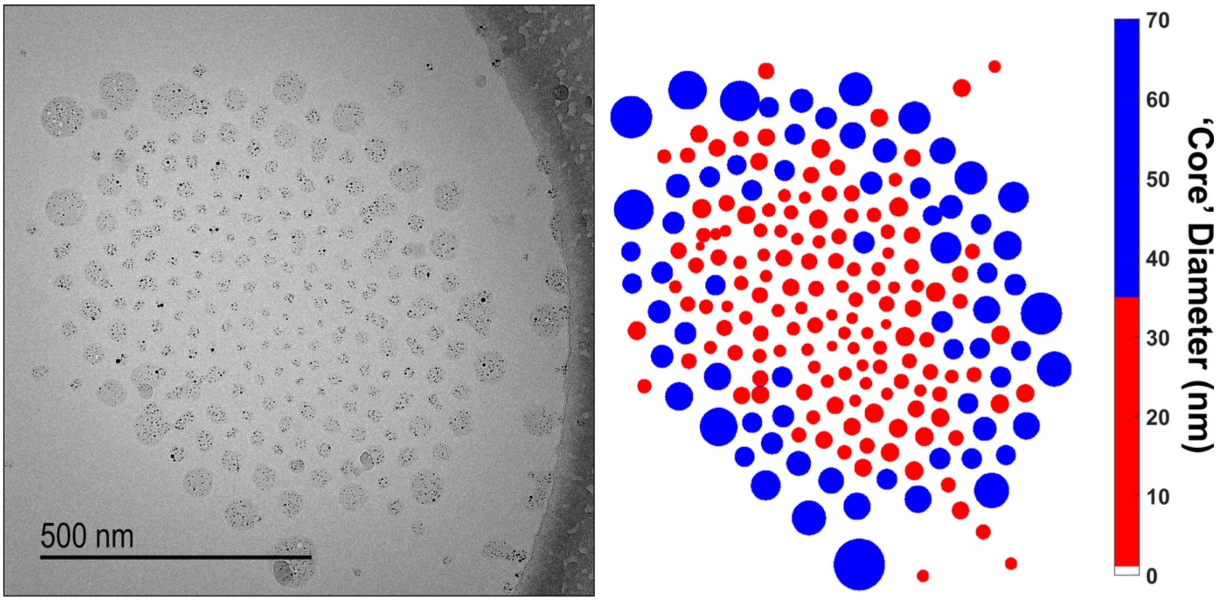

Figure S11. Left: cryoTEM micrograph of PAMAM-G5 dendrimicelles prepared using 1.5 times excess block copolymer. This off-stoichiometric amount results in a polydisperse dendrimicelle sample. Right: Color-coded heat map plot, showing the dendrimicelles with a core diameter (as indicated by the embedded gold nanoparticles) smaller than 35 nm in red, and the nanoaggregates with a 'core' diameter bigger than 35 nm in blue.

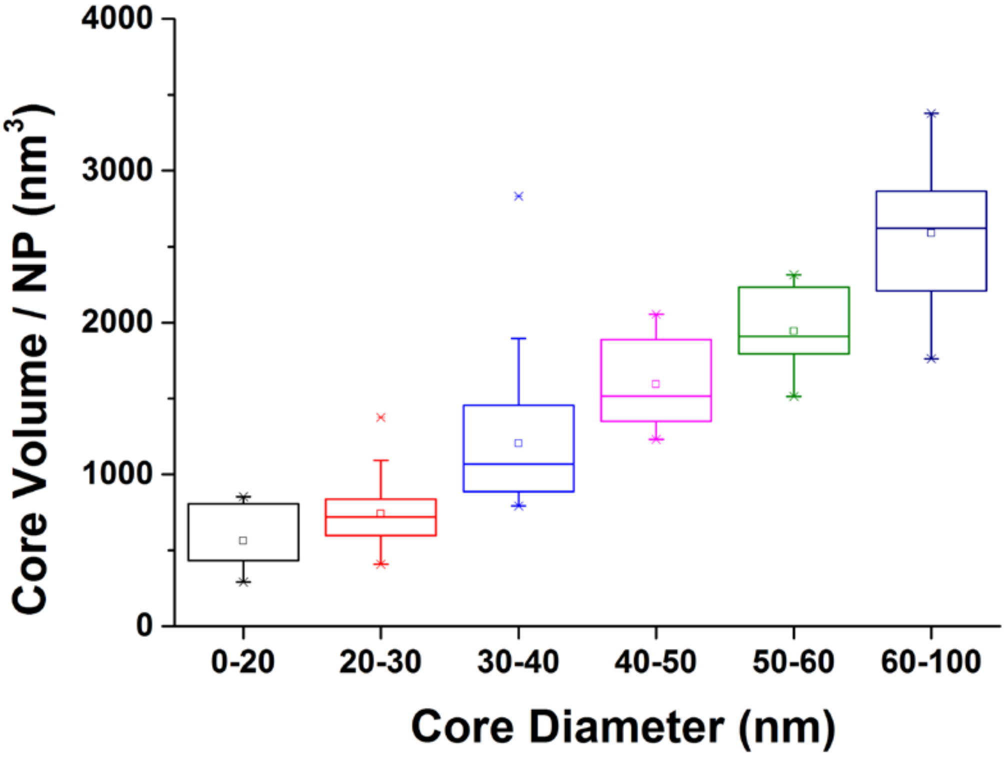

Figure S12. Plotting the volume-per-nanoparticle ratio also suggest the observed structures with apparent core diameters >30 nm to be vesicle-like structures. See also Figure 3 in the main text. The nanostructures with a core diameter in range 0-20 and 20-30 are not significantly different from each other (two-sample t-test, $p > 0.05$); all the other nanostructures are significantly different from each other ($p < 0.05$).

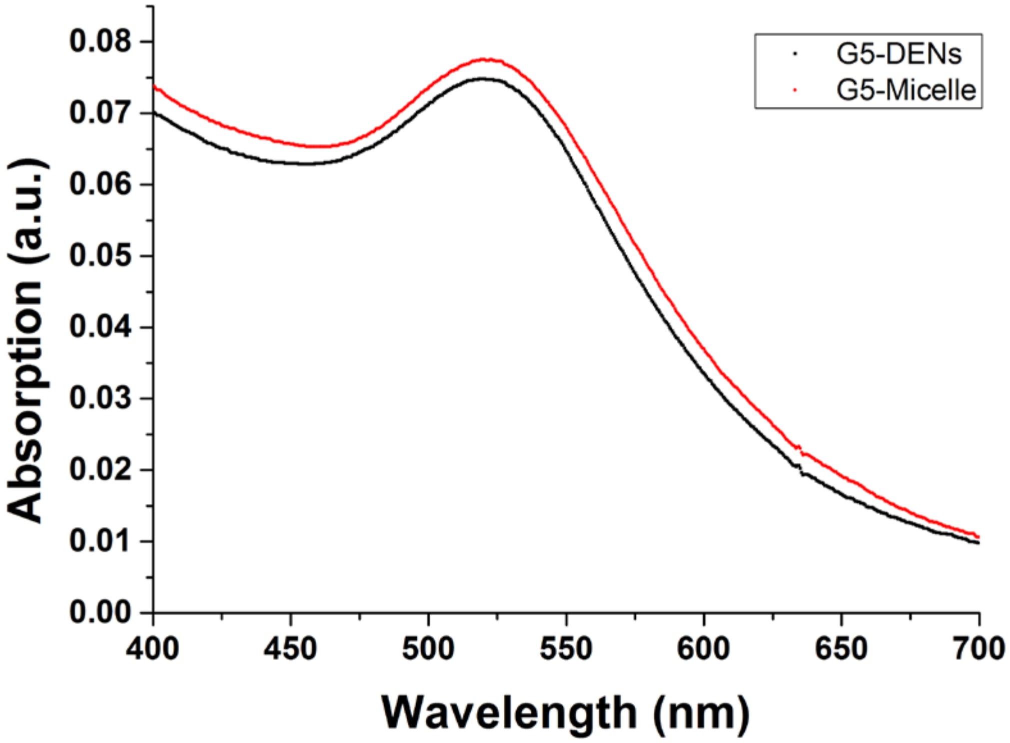

Figure S13 UV-Vis absorption plots of solutions of PAMAM-G5 DENs and corresponding dendrimicelles, recorded with exactly the same concentration of gold nanoparticles.
